# Stigma for common mental disorders in racial minorities and majorities a systematic review and meta-analysis

**DOI:** 10.1186/s12889-020-08964-3

**Published:** 2020-06-08

**Authors:** Ozlem Eylem, Leonore de Wit, Annemieke van Straten, Lena Steubl, Zaneta Melissourgaki, Gözde Topgüloğlu Danışman, Ralph de Vries, Ad J. F. M. Kerkhof, Kamaldeep Bhui, Pim Cuijpers

**Affiliations:** 1 Department of Clinical, Neuro and Developmental Psychology, Amsterdam Public Health Research Institute, Vrije Universiteit Amsterdam, Van der Boechorststraat 1, 1081 BT Amsterdam, The Netherlands; 2 Centre for Psychiatry, Queen Mary University of London, London, UK; 3 Department of Clinical Psychology and Psychotherapy, University of Ulm, Ulm, Germany; 4 Department of Psychology, University of Warwick, Coventry, UK; 5 Faculty of Social Sciences, Centre for Family and Couple Therapy, Özyeğin University, İstanbul, Turkey; 6 Medical Library, Vrije Universiteit, Amsterdam, the Netherlands

**Keywords:** Stigma, Mental illness stigma, Common mental disorders, Racial minorities

## Abstract

**Background:**

There is a strong stigma attached to mental disorders preventing those affected from getting psychological help. The consequences of stigma are worse for racial and/or ethnic minorities compared to racial and/or ethnic majorities since the former often experience other social adversities such as poverty and discrimination within policies and institutions. This is the first systematic review and meta-analysis summarizing the evidence on the impact of differences in mental illness stigma between racial minorities and majorities.

**Methods:**

This systematic review and meta-analysis included cross-sectional studies comparing mental illness stigma between racial minorities and majorities. Systematic searches were conducted in the bibliographic databases of PubMed, PsycINFO and EMBASE until 20th December 2018. Outcomes were extracted from published reports, and meta-analyses, and meta-regression analyses were conducted in CMA software.

**Results:**

After screening 2787 abstracts, 29 studies with 193,418 participants (*N* = 35,836 in racial minorities) were eligible for analyses. Racial minorities showed more stigma than racial majorities (g = 0.20 (95% CI: 0.12 ~ 0.27) for common mental disorders. Sensitivity analyses showed robustness of these results. Multivariate meta-regression analyses pointed to the possible moderating role of the number of studies with high risk of bias on the effect size. Racial minorities have more stigma for common mental disorders when compared with majorities. Limitations included moderate to high risk of bias, high heterogeneity, few studies in most comparisons, and the use of non-standardized outcome measures.

**Conclusions:**

Mental illness stigma is higher among ethnic minorities than majorities. An important clinical implication of these findings would be to tailor anti-stigma strategies related with mental illnesses according to specific racial and/or ethnic backgrounds with the intention to improve mental health outreach.

## Background

Common mental disorders (CMDs) such as depression and anxiety disorders are highly prevalent, disabling and costly with diminished quality of life, medical morbidity and mortality [1–3]. It is estimated that every year almost one in five people among the general population worldwide suffers from CMDs [4, 5]. Even though many people are affected by CMDs globally, there is a strong stigma attached to CMDs and those who have them [6]. Mental illness stigma is a multidimensional problem causing great burden on those who are affected [6, 7]. Not only does it determine negative public opinion and discrimination against people with mental illnesses [7] but it also leads to not to seek or adequately participate in psychological treatment [8–10].

There are various definitions of mental illness stigma in the current stigma literature. Recently, the Mental Illness Stigma Framework (MISF) has been proposed [11] emphasising that how stigma is experienced differs depending on the perspective of the general public who often attributes stigma (i.e. *stigmatizer*) to those who have mental illnesses (i.e. *stigmatized*) [11]. There are also shared perceptions and attributions between stigmatizer and stigmatized (i.e. *perceived stigma*). Different cognitive, affective and behavioural mechanisms are associated with each of these perspectives. The cognitive mechanisms are *stereotypes* referring to the collectively agreed upon negative beliefs about an individual with a mental illness (e.g. dangerousness, weakness) [11]. The affective mechanisms are *prejudice*s which are emotional reactions generated by stereotypes such as fear, anger and pity. The behavioural mechanisms are named as *discrimination* such as withholding help, avoidance, segregation or coercion [11]. The impact of stigma on an individual’s life can be understood in terms of three components: 1) *Experienced stigma,* referring to the day-to-day experiences of stereotypes, prejudice and discrimination from others, 2) *anticipated stigma*, the expectation to be a target of a stereotype, prejudice or discrimination, and 3) *internalised stigma*, which is the application of mental illness stigma to oneself such as believing that they are dangerous to others or they are incompetent [11].

There are differences in the extent of the impact of mental illness stigma depending on the racial and/or ethnic background of those who are affected [11]. Early research on the influence of ethnicity on the mental illness stigma indicated that compared to the White group, the non-White group perceived someone with mental illness as more dangerous [12] and expressed greater need for segregation than the White group [12]. These results were replicated by more recent research comparing Asian Americans [13–16], African Americans [17, 18] and Hispanics [19–21] with European Americans (White). Further, the variation in mental illness stigma could be even more so among specific ethnic groups within broad racial categories [22]. For instance, in a large scale study representative of the ethnic groups in Singapore, Subramaniam and colleagues found that, those of Indian ethnicity who also had low socio-economic status, perceived individuals with mental illness as more dangerous and unpredictable, and desired more social distance compared to those of Malay and Chinese backgrounds [22]. Thus, there is intersectionality in experiences of stigma [23, 24]. This perspective emphasises that the consequences of stigma are worse for some racial and/or ethnic groups who have for instance, personal incompetence attributions of mental illness [23] and who also face with other forms of “minority stress” and adversities such as interpersonal and structural discrimination within policies and institutions and low socio-economic background [8–10, 23, 24].

To date, there are no prior meta-analyses investigating racial and/or ethnic differences in mental illness stigma. This could be due to the inconsistency in how stigma mechanisms have been defined and measured [11]. One implication of this gap in the literature is the lack of information about the evidence base for developing culturally-relevant anti-stigma interventions [25]. Since CMDs are highly prevalent globally, it is important that psycho-social interventions focus on changing the negative stereotypes (e.g. I am incompetent) and/or discriminatory behaviours (e.g. social withdrawal) related with CMDs among racial and ethnic minorities specifically [25, 26]. Nevertheless, there are promising theoretical developments in the stigma field such as the MISF. There is also growing number of studies examining the ethnic variations in stigma for CMDs in Western as well as in Non-Western countries. Thus, the objectives of the current study are twofold. We examine the differences in mental illness stigma between racial minorities and majorities. We expect that racial minorities have more mental illness stigma for CMDs compared to majorities. We also investigate if there are variability in mental illness stigma between racial minorities depending on race, quality of the studies and types of stigma outcomes (self-report vs vignette).

## Methods

### Identification and selection of studies

#### Search strategy

A review protocol was developed based on the Preferred Reporting Items for Systematic Reviews and Meta-Analysis (PRISMA)-statement (www.prisma-statement.org) [27]. The protocol for this meta-analysis was registered at PROSPERO (CRD42018091080).

**Fig. 1:**
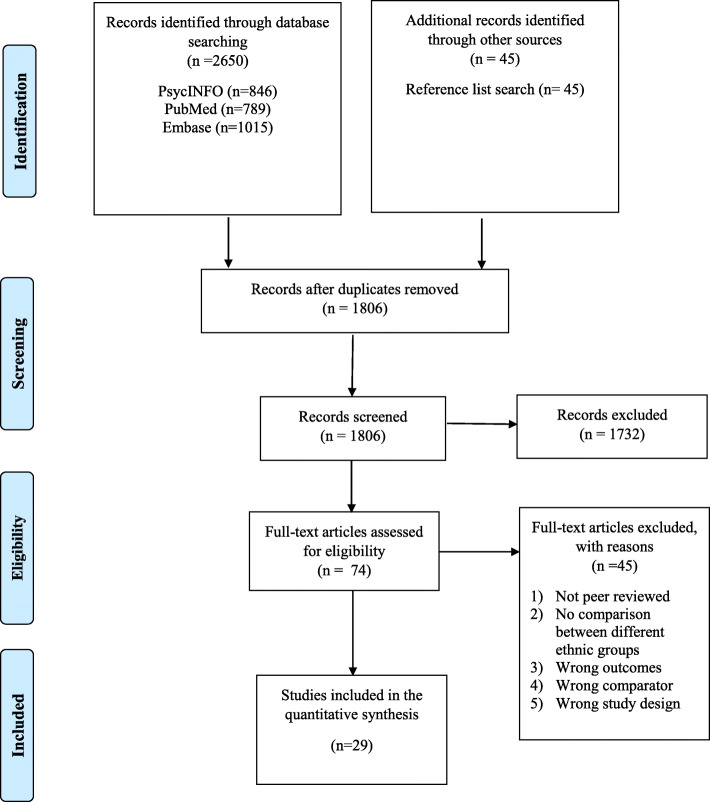
PRISMA flow chart of the study selection process

We conducted a comprehensive systematic literature search in the bibliographic databases PubMed, Embase.com and PsycINFO up to 20th December 2018, in collaboration with a medical librarian. Detailed search strategies for these databases are given in the Appendix 1. The following terms were used (including synonyms and closely related words) as index terms or free-text words: “Social stigma”, “Common mental disorders”, “Mood disorders”, “Anxiety disorders”, “Depression”, “Stress disorders”, “Migrants”, “Minority groups”. The search was performed without date or language restriction. Search strategies for other databases were built accordingly. We also checked the references of the included studies to identify additional relevant studies (see Fig. 1 for the Prisma Flowchart).

#### Inclusion criteria

The searches were limited to the following criteria: 1) Peer-reviewed papers, 2) Racial minorities (i.e. defined based on the classification of the country of the included studies) 2) Racial majorities (i.e. defined based on the classification of the country of the included studies) 3) Adults aged 18 and above and 4) Participants with/without common mental disorders (i.e. common mental disorders are identified as depression and anxiety spectrum disorders), 2) Empirical studies with cross-sectional designs measuring mental illness stigma about common mental disorders among racial minorities in comparison to majorities 3) Studies were not limited to the European populations only and studies carried out in other continents were included.

#### Exclusion criteria

The exclusion criteria: 1) Publications focusing on stigma about help-seeking, HIV, physical disorders or sexual minorities (if they are not from a racial minority group), 2) Publications focusing on stigma about severe mental health disorders (e.g. schizophrenia), 3) Empirical studies without a comparison group (studies which are not comparing different racial groups were excluded), 4) Qualitative studies and 5) Publications focusing on adolescent and children sample.

### Quality assessment

We assessed the quality of the included studies using the Effective Public Health Practice Project Quality Assessment Tool (EPHPP). This tool assesses possible sources of bias in observational studies and RCTs. Since we have included cross-sectional studies, the following domains of the tool were used in this study: (1) selection bias; (2) study design; (3) confounders; (4) data collection method and; (5) analyses. The studies received an overall assessment in one of the following: 1) high risk of bias (studies which scored high risk of bias in 3 or more of the assessment domains); 2) moderate risk of bias (studies which scored high risk of bias in 2 of the assessment domains) and; 3) low risk of bias (studies which scored high risk of bias in 1 of the assessment domains). Assessment was carried out by two independent assessors and disagreements were solved through discussions.

### Data extraction

A customised data extraction form was generated and included the following characteristics: Method of recruitment into the study (community, clinical samples or other recruitment type), target group (adults in general, older adults, student population or other target group), types of stigma perspectives, types of stigma mechanisms and types of outcome measures used to measure stigmas (self-report instruments, vignettes). Vignettes are case descriptions of an individual, presenting symptoms of a CMD [22] (see Appendix 2 for an example).

Covidence, online software for screening and data extraction for systematic reviews and meta-analyses, was used for the review and extracting data. First, the results of the online database searches were imported to covidence. Two reviewers had personal accounts and selected papers independently in a random order. A third reviewer carried out the reference list search of the selected papers. The titles of all studies were screened, and the abstracts of the studies were checked regarding the inclusion criteria. When no definitive decision could be made based on the abstract, the original papers were used. Discrepancies between the reviewers’ selections were resolved through discussions. If not resolved, the opinion of a fourth researcher was sought. The corresponding author filled in the extraction form.

### Conceptual frameworks

We conceptualised mental illness stigma based on the MISF [11]. The types of perspectives, measured by the studies, were categorised into three groups: 1) the perspective of the stigmatizer (i.e. Public attitudes and beliefs that other people devalue or discriminate against individuals with mental illness. The specific components are: stereotype, prejudice and discrimination); 2) The perspective of the stigmatized (i.e. Personal beliefs, attitudes and perceived anger for having a mental illness. The specific components are: experienced stigma, anticipated stigma and internalized stigma) and; 3) The perceived stigma (i.e. shared experiences of stereotypes, prejudices and discrimination between people who stigmatize and who are stigmatized).

The concept of race-ethnicity was defined based on the minority or majority classifications of the country of the included studies. Because there were many ethnic groups (e.g. Chinese, Indian) within the included studies, we decided to use broad racial categories (e.g. Asian, Black) in order to make the studies comparable [28]. Six racial groups were identified in consultation with the categories previously defined by Ünlü İnce and colleagues [29]. These categories were: Black (African background), Asian, Hispanic (Latin American and Spanish background), Native American (referring to the indigenous people of North America), and White (Caucasian and white European) and other (people from racial-ethnic minority group who could not be identified in one of these categories).

### Outcome

For each comparison between ethnic groups in stigma, the effect size indicating the difference between groups was calculated (Hedge’s g). Effect size of 0.8 was accepted as large, effect size of 0.5 was accepted as moderate and effect size of 0.2 was accepted as small [30]. Effect sizes were calculated by subtracting the means of stigma between racial minorities and majorities and dividing the result by the pooled standard deviation. If means and standard deviations were not reported, we used the procedures of the Comprehensive Meta-Analysis software (see below) to calculate the effect size using dichotomous outcomes; and if these were not available either, we used other statistics (such as t-value or *p*-value) to calculate the effect size.

In order to calculate effect sizes we used all the self-report measures and vignettes examining mental illness stigma (see Table 1 for the outcome measures) [such as Perceived Devaluation and Discrimination Scale (PDD) [48], Internalized Stigma of Mental Illness Scale (ISMI) [49], Community Attitudes to Mental Illness scale (CAMI) [33]]. The decision on which outcome measure is capturing which specific stigma perspective and mechanism was based on the Fox and colleagues` classification [11].

**Table 1:** Selected characteristics and main findings of cross-sectional studies comparing ethnic minorities with majorities on stigma outcomes (*N* = 29)

Study	Country	Type of CMD	Racial groups	Recruitment	Outcomes	Results
Adewuya, 2008 [31]	Nigeria	Various	Black (N_maj_ = 1869) Other (N_min_ = 92)	Community	• Self-report • SDS	• There were no ethnic differences in discrimination against people with mental illness (*p* = 0.14)
Ahn 2015 [32]	North Korea	Various	Asian (N_maj_ = 3055) Asian (N_min_ = 545)	Other	• Self-report • PDD	• Asian majorities (M = 35.64) had higher perceived stigma than Asian minorities (M = 37) (*p* = 0.007)
Anglin 2006 [18]	USA	Various	White (N_maj_ = 913) Black (N_min_ = 118)	Community	• Vignette • Study-constructed	• There was more stereotype (i.e. perceived dangerousness) against people with depression among Black group compared to White group (t = 2.14) (*p* < .05) • There was less prejudice (i.e. tendency to blame) against people with depression among Black group compared to White group (*t* = − 2.33) (*p* < .05) • There was less discrimination (i.e. tendency to endorse punishment) against people with depression among Black group compared to White group (*t* = − 3.91) (*p* < .001)
Aznar-Lou 2016 [33]	Catalonia, Spain	Various	White (N_maj_ = 1668) White (N_min_ = 56) Black (N_min_ = 58) Asian (N_min_ = 7) Other (N_min_ = 82)	Community	• Self-report • CAMI-23	• Asian (M = 20.9), Other (M = 23.0) Black (M = 23.5) and White minority (M = 23.9) had less stereotypes (i.e. less favourable attitudes in authoritarianism) against those with CMDs) compared to White majority (M = 25.0) • Asian (M = 25.4) Other (M = 26.9) and Black (M = 27.0) groups had less stereotypes (i.e. favourable attitudes in benevolence) compared to white minority (M = 27.8) and majority (M = 27.7) • Asian (M = 34.3) Other (M = 36.2) Black (M = 36.4) had less stereotypes (i.e. favourable attitudes toward supporting those with CMDs) compared to White minority (M = 37.3) and majority (M = 37.5) • Asian group (M = 13.7) had more discrimination (i.e. least favourable attitudes towards those with CMDs) compared to Other (M = 15.3) Black (M = 15.5) White minority (M = 16.6) majority (M = 16.5)
Brown 2010 [19]	USA	Various	White (N_maj_ = 229) Black (N_min_ = 220)	Community	• Self-report • ISMI • PDD	• There were no differences between Black (M = 31.3, SD = 4.1) and White (M = 31.0, SD = 4.8) groups in perceived stigma (*p* = .55) • There were no differences between Black (M = 65.9, SD = 11) and White (M = 65.0, SD = 11.4) groups in internalised stigma (*p* = .42)
Caplan 2011 [22]	USA	Depression	Hispanic (N_maj_ = 91) Hispanic (N_min_ = 86)	Clinical	• Self-report • Study-constructed	• There was higher anticipated stigma among minority Hispanic group compared to the majority (*p* = 0,015)
Cheng 2015 [15]	USA	Depression	White (N_maj_ = 206) Asian (N_min_ = 231)	Community	• Vignette • AQ	• There was more discrimination(i.e. desire for social distance) against a person with depression among Asian group (M = 3.16, SD = 1.06) compared to White (M = 2.80, SD = 1.18) *p* = 0.004 • There was more discrimination (i.e. less willingness to hire and rent) against a person with depression among Asian group (M = 5.54, SD = 1.64) compared to White (M = 6.00, SD = 1.82) *p* = 0.008 • There was more prejudice (i.e. blame) against a person with depression among Asian group (M = 4.18, SD = 1.63) compared to White (M = 3.73, SD = 1.78) *p* = 0.02 • There was more prejudice (i.e. anger) against a person with depression among Asian group (M = 3.26, SD = 1.90) compared to White (M = 2.58, SD = 1.72) *p* = 0.002 • There was more prejudice (i.e. fear of someone) against someone with depression among Asian group (M = 3.78, SD = 1.96) compared to White (M = 3.32, SD = 2.01) *p* = 0.54
Conner 2010 [34]	USA	Various	White (N_maj_ = 229) Black (N_min_ = 201)	Community	• Self-report • ISMI • PDD	• There was no differences in perceived stigma between Black (M = 2.61, SD = 0.28) and White (M = 2.59, SD = .29) groups t [246] = − 0.58 • There was more internalised stigma among Black (M = 2.18, SD = 0.30) compared to White (M = 2.10, SD = 0.30) group (t [246] = − 2.118, *p* = .035).
Conner 2009 [35]	USA	Various	White (N_maj_ = 51) Black (N_min_ = 48)	Other	• Self-report • ISMI • PDD	• There was more perceived stigma among Black (M = 2.90, SD = 0.75) compared to White (M = 2.32, SD = 0.55) group (*p* < .001) • There was more internalised stigma among Black (M = 2.75, SD = 0.81) compared to White (M = 2.30, SD = 0.53)
Copelj 2011 [36]	Australia	Depression	White (N_maj_ = 54) Other (N_min_ = 54)	Community	• Self-report • DSS	• There was more perceived stigma (i.e. perceived attitudes of others about depression) among Other group (M = 17.82, SD = 7.58) compared to White (M = 9.03, SD = 5.36) *F* = 32.95 • There was more stereotype (i.e. personal attitudes toward depression) about depression among Other group (M = 25.16, SD = 6.13) compared to White (M = 19.35, SD = 8.79) *F* = 10.78
Eisenberg 2009 [37]	USA	Various	White (N_maj_ = 3780 Asian (N_min_ = 579) Black (N_min_ = 266) Hispanic (N_min_ = 302) Combination (N_min_ = 240) Other (N_min_ = 290)	Students	• Self-report • PDD	• There was more perceived stigma for depression among Black (M = 2.77),Hispanic (M = 2.50) Asian (M = 2.50), Combination (M = 2.48) and Other (M = 2.54) groups compared to White (M = 2.38 • There was more stereotype (i.e. personal attitudes toward depression) about depression among Asian (M = 1.45) compared to Black (M = 0.93), Hispanic (M = 1.05), Combination (M = 0.91), Other (M = 1.10) groups compared to White (M = 0.95)
Fogel 2005 [16]	USA	Depression	White (N_maj_ = 66,817) Asian (N_min_ = 1839)	Community	• Self-report • Study-constructed	• There was more anticipated stigma for depression related with depression among Asian (M = 2.45, SD = 1.22) compared to White (M = 2.10, SD = 1.25) *F* = 144.40, (*p* < 0.001) • There was more anticipated stigma for depression related with employer among Asian (M = 2.93, SD = 1.07) compared to White (M = 2.68, SD = 1.16) *F* = 85.55, (*p* < 0.001) • There was more anticipated stigma for depression related with family among Asian (M = 2.23, SD = 1.19) compared to White (M = 1.71, SD = 1.18) *F* = 360.38 (*p* < 0.001)
Georg Hsu 2008 [17]	USA	Depression	White (N_maj_ = 100) Asian (N_min_ = 100)	Community	• Vignette • Study-constructed	• There was more stereotype (i.e. personal attitudes toward depression) about depression among Asian (M = 39.4) compared to White (M = 15.0) *P* = 0.000
Givens 2007 [21]	USA	Depression	White (N_maj_ = 68,319) Black (N_min_ = 3596) Asian (N_min_ = 2794) Hispanic (N_min_ = 3203) Other (N_min_ = 841)	Community	• Self-report • Study-constructed	• There was more anticipated stigma for depression related with family among Asian (M = 71.7, SD = 1.24), Black (M = 68.5, SD = 1.24), Hispanic (M = 61.8, SD = 0.89) and Other (M = 60.4, SD = 0.96) groups compared to White (M = 63.1, SD = 1.00) • There was more anticipated stigma for depression related with family among Asian (M = 55.0, SD = 1.30), Black (M = 45.4, SD = 1.08), Hispanic (M = 42.8, SD = 0.91) and Other (M = 43.01, SD = 1.01) groups compared to White (M = 43.03, SD = 1.0) • There was more anticipated stigma for depression related with employer among Asian (M = 42.9, SD = 1.88), African (M = 26.8, SD = 0.92), • Hispanic (M = 28.1, SD = 0.96) and Other (M = 27.5, SD = 1.01) groups compared to White (M = 27.9, SD = 1.0)
Hickie 2007 [34]	Australia	Depression	White (N_maj_ = 38) Asian (N_min_ = 184)	Students	• Self-report • Study-constructed	• There were no differences in discrimination against those with depression related with employer among Asian compared to White groups (*p* = 1.00) • There were no differences in discrimination against those with depression related with family (among Asian compared to White groups (*p* = 0.05) • There was more discrimination against those with depression related with friends among Asian compared to White groups (*p* = 0.04) • There was more discrimination against those with depression related with doctor/health professional among Asian compared to White groups (*p* = 0.001) • There was more stereotype (i.e. perception of those with depression as dangerous) among Asian compared to White groups (*p* = 0.000) • There was more prejudice (i.e. blame) against those with depression among Asian compared to White groups (*p* = 0.000)
Jimenez 2012 [23]	USA	Various	White (N_maj_ = 1257) (Black (N_min_ = 536) Asian (N_min_ = 112) Hispanic (N_min_ = 303)	Other	• Self-report • Study-constructed	• There was more anticipated stigma for having any CMDs among Hispanic (40.3%) compared to Asian (25.9%), Black(12.9%) groups compared to White (15.3%) *p* = 0.000
Makowski 2017 [11]	Germany	Various	White (N_maj_ = 1622) Other (N_min_ = 364)	Community	• Self-report • Study-constructed	• Other group had more prejudice (i.e. perception of migrants with depression as scary) (M = 2.28; SE = 0.11) compared to White group (M = 1.82; SE = 0.04)*F* = 8.179; (*p* = 0.000) • Other group had more prejudice (i.e. perception of migrants with depression as having problems with comprehension) (M = 2.04, SE = 0.11) compared to White groups (M = 1.64, SE = 0.04) *F* = 5.796, (*p* = 0.003) • Other group had more prejudice (i.e. feeling more uncomfortable) (M = 2.50, SE = 0.13) against migrants with depression compared to White (M = 2.00; SE = 0.04) *F* = 9.339 (*p* = 0.000) • Other group had more stereotypes (i.e. perception of migrants with depression as feeling inadequate around others) (M = 2.47, SE = 0.07) compared to White (M = 2.31, SE = 0.02) *F* = 3.539 (*p* = 0.029)
Menke 2009 [38]	USA	Depression	White (N_maj_ = 744) Black (N_min_ = 147)	Clinical	• Self-report • LSCS	• There was more perceived stigma for depression among Black group (M = 46.16; SD = 12.59) compared to White (M = 41.95; SD = 18.89) t = 3.35 (*p* = 0.000)
Mokkarala 2016 [39]	USA	Various	White (N_maj_ = 116) Asian (N_min_ = 61)	Students	• Self-report • Study-constructed	• There were no significant differences in perceived stigma (shame) for having any CMDs between White (M = 1.90, SD = 0.67) and Asian groups (M = 2.04, SD = 0.57), t = 1.29
Nadeem 2007 [8]	USA	Depression	White (N_maj_ = 886) Black (N_min_ = 1497) Hispanic (N_min_ = 5153)	Clinical	• Self-report • Study-constructed	• There was more stereotype (i.e. personal attitudes toward depression) about depression among Black (*p* = .037) and Hispanic (*p* = .30) groups compared to White.
O’Mahen 2011 [40]	USA	Depression	White (N_maj_ = 251) Black (N_min_ = 281)	Other	• Self-report • LSCS	• There was more perceived stigma for depression among Black (M = 42.31, SD = 5.76) compared to White groups (M = 40.04, SD = 6.44) (*p* = 0.000)
Papadopoulos 2002 [41]	UK	Various	White (N_maj_ = 79) Other (N_min_ = 91)	Community	• Self-report • CAMI-23	• There was more discrimination (i.e. desire for more social distance) towards those with CMDs among Other group compared to White (*p* < .001) • There was more stereotype about those with CMDs among Other group compared to White (*p* < .001)
Picco 2016 [42]	Singapore	Various	Asian (N_maj_ = 150) Asian (N_min_ = 130)	Clinical	• Self-report • ISMI	• There was more internalised stigma (i.e. alienation, social withdrawal) among the minority Asian group compared to the majority (*p* = 0.615); IN (*p* = 0.161)
Rao 2007 [43]	USA	Various	White (N_maj_ = 158) Black (N_min_ = 71) Asian (N_min_ = 28) Hispanic (N_min_ = 100)	Students	• Vignette • AQ	• There was more stereotype (i.e. perceiving people with CMDs and dangerous) among Black (M = 14)(*p* < .001) and Asian (M = 11) groups compared to White (M = 12) and Hispanic (M = 9) (*p* < .001) • There was more discrimination (i.e. desire for segregation) against those with CMDs among African (M = 13) (*p* < .001), Asian (M = 13) groups compared to White (M = 11) and Hispanic (M = 10) (*p* < .005)
Rüsh 2012 [44]	UK	Various	White (N_maj_ = 2990) Comb (N_min_ = 429)	Community	• Self-report • CAMI-23	• There was more prejudice and discrimination (i.e. desire for segregation) against people with CMDs among Black (*p* < 0.001) and Asian groups compared to White (*p* < 0.001) • There was less tolerance and support for people with CMDs among Black (*p* < 0.001) and Asian groups compared to White (*p* = < 0.005) • There was more discrimination against those with CMDs among African (*p* < 0.001) and Asian groups compared to White (*p* < 0.001)
Schafer 2011 [45]	UK	Various	White (N_maj_ = 209) Black (N_min_ = 63)	Students	• Self-report • CAMI-23	• There was more stereotype (i.e. negative attitudes) against those with any CMDs among Black (M = 2.27) compared to White groups (M = 1.93) t = − 4.563 (*p* = 0 < 001)
Shamblaw 2015 [46]	Canada	Depression	White (N_maj_ = 200) Asian (N_min_ = 276)	Students	• Self-report • DAQ • SDS	• There was more stereotype against those with depression among Asian (M = 115.71, SD = 24.74) compared to White (M = 105.72, SD = 27.08), t = 4.07 (*p* < 0.001) • There was more discrimination (i.e. desire for social distance) among Asian (M = 37.30, SD = 9.21) compared to White groups (M = 40.26, SD = 9.40), t = 3.34, (*p* = 0.001)
Subramaniam 2017 [23]	Singapore	Various	Asian (N_maj_ = 1034) Asian (N_min_ = 977) Asian (N_min_ = 963) Other (N_min_ = 32)	Community	• Self-report • DSS	• There was more discrimination (i.e. desire for social distance) against those with CMDs among majority Asian group (M = 12.00, SE = 0.09) compared to minority Asian groups (M = 10.89, SE = 0.09), (M = 11.52, SE = 0.11) and Other (M = 11.71, SE = 0.45) (*p* < .001) • There was more perceived stigma (i.e. perception of those with CMDs as weak not sick) among minority Asian groups (M = 10.95, SE = 0.06), (M = 10.74, SE = 0.08) compared to the majority (M = 10.07, SE = 0.06) (*p* < .001) • There was more perceived stigma (i.e. perception of those with CMDs as dangerous and unpredictable) among minority Asian groups (M = 11.60, SE = 0.09),(M = 11.75, SE = 0.11) compared to the majority (M = 11.61, SE = 0.08) (*p* = 0.66)
Wang 2013 [47]	USA	Various	White (N_maj_ = 467) Black (N_min_ = 221) Hispanic (N_min_ = 57) Other (N_min_ = 65)	Students	• Vignette • SDS	• There was more discrimination (i.e. desire for social distance) against those with any CMDs among Black (M = 24.28, SD = 5.04), Other (M = 23.60, SD = 6.23) and Hispanic (M = 23.17, SD = 4.87) compared to White (M = 22.41, SD = 5.07), *F* = 6.32 (*p* = 0.000)

*Various* Several CMDs are studied together and/or the type of CMD was not specified, *CMDs* Common Mental Disorders, Recruitment: *Community* Community sample, *Clinical* Clinical sample, *Student* Student sample, *AQ* Attribution Questionnaire, *CAMI-23* Community Attitudes towards Mentally Ill Scale, *ISMI* Internalized Stigma of Mental Illness Scale, *PDD* Perceived Devaluation and Discrimination Scale, *SDS* Social Distance Scale, *DSS* Depression Stigma Scale, *DAQ* Depression Attribution Questionnaire, *LSCS* Link Stigma Consciousness Scale, *Study-Constructed* study-constructed questionnaires, *N*_*min*_ Sample size for racial minorities, *N*_*maj*_ Sample size for racial majorities, *M* mean, *SD* standard deviation, *p p* value, *SE* standard error, *t* t statistic, *F* F statistic

### Analyses

To calculate pooled mean effect sizes, we used the computer programme Comprehensive Meta-Analysis (version 3.3070; CMA). We expected considerable heterogeneity among the studies for various reasons. First, our definition of racial minorities and majorities were too broad and did not capture specific ethnic minorities and majority groups within the samples. Second, we pooled the studies employing different outcome measures (self-report and vignette). Third, the included studies are investigating different stigma perspectives and mechanisms associated with them. Fourth, the included studies often investigated mental illness stigma for various common mental illnesses (both anxiety and depression spectrums) and lastly, we included studies from both High Income and Low and Middle Income countries which have variations in how mental illness stigma is defined, measured and experienced. In the light of these, we employed a random effects pooling model in all analyses.

As a test of homogeneity of effect sizes, we calculated the *I*^*2*^ statistic, which is an indicator of heterogeneity in percentages. A value of 0% indicates no observed heterogeneity, and larger values indicate increasing heterogeneity, with 25% as low, 50% as moderate and 75% as high heterogeneity [50]. We calculated 95% confidence intervals around *I*^*2*^ [51] using the non-central chi-squared based approach within the heterogi module for Stata [52].

We tested publication bias by inspecting the funnel plot on primary outcome measures and by Duval and Tweedie’s trim and fill procedure [53] yields an estimated effect size after publication bias has been taken into account (as implemented in CMA). We also conducted Egger’s test of the intercept to quantify the bias captured by the funnel plot and to test whether it was significant.

We also examined whether specific characteristics of the studies were related to the effect sizes. We conducted subgroup analyses according to the mixed effects model, in which studies within subgroups are pooled with the random effects model, while the tests for significant differences between subgroups are conducted with the fixed effects model. The priori decided sub-groups were: ethnicity, type of stigma outcome, type of stigma perspective and the quality of the studies. Further, we used multi-variate meta-regression analyses as implemented in CMA.

## Results

### Selection and inclusion of the studies

After examining a total of 2787 abstracts (1806 after removal of duplicates), we retrieved 1806 full text papers for further consideration. We excluded 1732 of the retrieved papers. The PRISMA flowchart describing the inclusion process, including the reasons for exclusion, is presented in Fig. 1. A total of 29 studies were included in the quantitative synthesis.

### Characteristics of the included studies

Selected characteristics of the included studies are presented in Table 1. Of all the studies included in the analysis, (*N* = 18) 62% were conducted in the United States and (*N* = 3) 10% were conducted in the United Kingdom. The rest of the countries were: Singapore (*N* = 2) 6%; Australia (*N* = 2) 6%; Spain (*N* = 1) 3%; Nigeria (*N* = 1) 3%; Germany (*N* = 1) 3%; Canada (*N* = 1) 3% and; Korea (*N* = 1) 3%.

Regarding the participant characteristics, a total of 157,582 (81%) participants were from the majority racial groups and a total of 35,836 (19%) were from the minority racial groups. Minority and majority statuses were defined based on the country of the included study. Of all the participants from the racial majority groups, 96.06% (*N* = 151,383) were White, 2.69% (*N* = 4239) were Asian, 0.06% (*N* = 91) were Hispanic and 1.18% (*N* = 1869) were Black. Further, of all the participants from the racial minority groups, 11.30% (*N* = 4051) were Hispanic, 57.09% (*N* = 20,462) were Black, 23.11% (*N* = 8285) were Asian, 9.31% (*N* = 3338) were from other racial background.

With respect to the condition of the CMD, more than half of the studies (*N* = 17) investigated various CMDs and/or not specified the type of CMD in the study, whereas (*N* = 12) 41% studies investigated depression.

A considerable number of the studies (48%) recruited community sample (*N* = 14), 24% recruited student sample (*N* = 7), 14% recruited other sample (e.g. geriatric population) (*N* = 4) and 14% recruited participants from clinical populations (*N* = 4). Regarding the outcomes, (*N* = 7) 24% used a vignette approach to measure stigmas whereas, (*N* = 22) 76% used self-report questionnaires.

### Risk of bias

The risk of bias can be seen in Table 2. When taking into account the five different bias items, 20 studies (68%) were rated as high risk of bias, 6 studies (21%) were rated as moderately high risk of bias and only 3 (10%) studies were rated as low risk of bias. The selection bias was rated as low risk of bias in 10 studies (34%), high risk of bias in 18 studies (62%), and unclear in 1 study (3%). When taking into account the study design, there was a high risk of bias in 18 studies (62%) and low risk of bias in 11 studies (38%). Data collection methods were rated as low risk of bias in 10 studies (34%), high risk of bias in 16 studies (55%) and unclear in 3 studies (10%). The risk for confounders was high in 20 studies (68%) and low in 9 studies (31%). As for the data analyses, the risk of bias was low in 22 studies (76%), high in 1 study (3%) and unclear in 6 studies (21%) (see Table 2).

**Table 2:** Risk of Bias Assessment of all the studies (*N* = 29)

***Study***	***Type of Studies***	***Analyses***	***Confound***	***Data collection***	***Select Bias***	***Study Design***	***RoB***^**a**^
Adewuya, 2008 [31]	Self-report	Low	High	Low	Low	High	High
Ahn 2015 [32]	Self-report	Low	High	Low	Low	High	High
Anglin 2006 [18]	Vignette	Low	Low	High	Low	Low	High
Aznar-Lou 2016 [33]	Self-report	Low	Low	Low	High	Low	Moderate
Brown 2010 [19]	Self-report	High	Low	High	Low	Low	High
Caplan 2011 [22]	Self-report	Low	High	High	High	High	High
Cheng 2015 [15]	Vignette	Low	Low	Unclear	Low	Low	Moderate
Conner 2010 [34]	Self-report	Low	High	Low	Low	Low	Moderate
Conner 2009 [35]	Self-report	Low	High	Low	High	Low	High
Copelj 2011 [36]	Self-report	Low	High	High	High	Low	High
Eisenberg 2009 [37]	Self-report	Low	Low	Low	High	Low	Moderate
Fogel 2005 [16]	Self-report	Low	High	High	Low	Low	High
Georg Hsu 2008 [17]	Vignette	Unclear	High	High	High	High	High
Givens 2007 [21]	Self-report	High	High	Low	High	Low	High
Hickie 2007 [34]	Self-report	Low	High	High	High	Low	High
Jimenez 2012 [23]	Self-report	Low	Low	High	Unclear	Low	Moderate
Makowski 2017 [11]	Self-report	Low	Low	High	Low	Low	Moderate
Menke 2009 [38]	Self-report	Low	High	Low	High	High	High
Mokkarala 2016 [39]	Self-report	Low	High	High	Low	Low	High
Nadeem 2007 [8]	Self-report	Low	Low	High	High	Low	High
O’Mahen 2011 [40]	Self-report	Low	Low	Unclear	High	High	High
Papadopoulos 2002 [41]	Self-report	Low	High	Unclear	High	High	High
Picco 2016 [42]	Self-report	Low	Low	Low	High	High	High
Rao 2007 [43]	Vignette	Unclear	Low	Low	High	Low	High
Rüsh 2012 [44]	Self-report	Low	Low	Low	Low	Low	Low
Schafer 2011 [45]	Self-report	High	High	Low	High	High	Low
Shamblaw 2015 [46]	Self-report	Low	High	Low	High	High	High
Subramaniam 2017 [23]	Vignette	Low	Low	Low	Low	Low	Low
Wang 2013 [47]	Vignette	Low	High	High	High	High	High

*Low* Low risk of bias, *High* High risk of bias, *Unclear* reviewers were not able to reach consensus due to lack of information; ^a^n this column, high refers to the high risk of bias (studies which scored high risk of bias in 3 or more of the assessment domains); moderate refers to the moderate risk of bias (studies which scored high risk of bias in 2 of the assessment domains) and low refers to the low risk of bias (studies which scored high risk of bias in 1 of the assessment domains); *RoB* Risk of Bias Assessment

We assessed whether the authors used stratification and/or matching in the study design in order to control possible confounders [54, 55]. Only 4 studies (14%) used stratification. Further, the representativeness of the samples was also limited as only 4 studies (14%) used random sampling, whereas the rest of the studies used convenience sampling. In one study, the authors recruited participants from their friends and networks of their friends which increased the chances of selection bias and restricted the representativeness of the racial groups in their sample [15].

Out of the 21 self-report studies, 7 (33%), and out of the 8 vignette studies, 3 (37%) used “study constructed” questionnaires (i.e. questionnaires which are constructed by the author for the purpose of the study) as outcome measures. The psychometric properties of these questionnaires were not tested.

### Mental illness stigma for CMDs between racial minorities and majorities

First, we run the analyses separately for the studies investigating depression only g = 0.22 (95% CI: 0.10 ~ 0.34) (*I*^2^ = 94, 95% CI: 93 ~ 96) and for the studies investigating various types of CMDs together g = 0.18 (95% CI: 0.10 ~ 0.28) (*I*^2^ = 86, 95% CI: 81 ~ 90). Since this resulted with small effect sizes and with very high heterogeneity in each, we decided to pool all the studies together, regardless of the condition of the mental illness studied, for further sensitivity analyses.

**Fig. 2:**
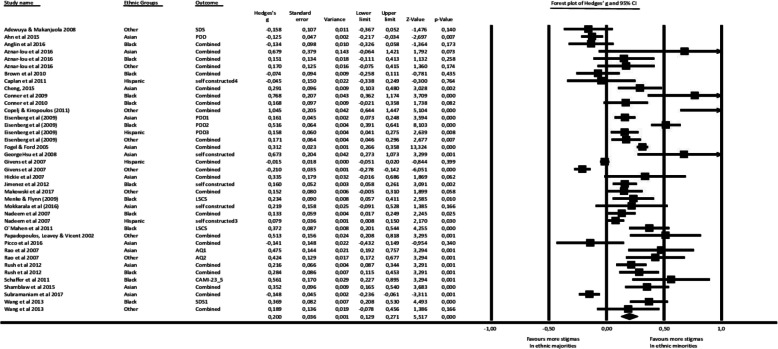
Stigma for CMDs between ethnic minorities and majorities: Forest plot for all studies. Not. The combined refers to the same outcomes which are automatically pooled together by the CMA software when the authors run the analyses. For instance, if different studies used CAMI questionnaire and measured stereotypes among Black racial group, they are pooled together by the CMA

Primary outcome was mental illness stigma defined by the MISF [11]. Cognitive (e.g. stereotype), affective (e.g. prejudice), behavioural (e.g. discrimination) and/or combination of each of these components of mental illness stigma were compared between racial minorities and majorities in 29 studies (39 comparisons). We have decided to pool the studies together as stigmatizer perspective measuring the cognitive component of the stigma was over represented, whereas stigmatized perspective measuring how stigma was anticipated was under represented (see Table 1). The overall effect size was small but significant g = 0.20 (95% CI: 0.12 ~ 0.27) indicating that racial minorities had more mental illness stigma about CMDs when compared with the majorities, with very high heterogeneity (*I*^2^ = 91, 95% CI: 89 ~ 93). The results of the analyses are reported in Table 3, and the forest plot is given in Table 4 (see figure 2 in Appendix 3 for the forest plot) .

**Table 3:** Stigma for racial minorities and majorities: Pooled effect sizes of primary outcomes

*Characteristics*	*N*_comp_	*g*	95% *CI*	*I*^2^	95% *CI*	*P*^*a*^
Primary Analyses						
All analyses	39	0.20	0.12 ~ 0.27	91%	89 ~ 93	**<.001**
High risk of bias studies excluded	29	0.20	0.10 ~ 0.25	88%	84 ~ 90	**<.001**
Standardized outcomes only	16	0.23	0.10 ~ 0.36	84%	75 ~ 89	**<.001**
Outliers excluded	12	0.29	0.21 ~ 0.36	29%	0 ~ 63	**<.001**
Subgroup Analyses						
Ethnicity Asian	4	0.29	0.17 ~ 0.42	23%	0 ~ 75	**<.001**
Black	5	0.30	0.13 ~ 0.36	55%	0 ~ 81	**<.001**
Other	3	0.26	− 0.01 ~ 0.41	17%	0 ~ 77	**. 001**
Total between^b^						.93
Quality Moderate	5	0.21	0.10 ~ 0.32	0%	0 ~ 64	**<.001**
Strong	2	0.24	0.13 ~ 0.34	a)	b)	**<.001**
Weak	5	0.40	0.26 ~ 0.54	33%	0 ~ 75	**<.001**
Total between						.08
Outcome Self-report	7	0.25	0.14 ~ 0.36	37%	0 ~ 72	**<.001**
Vignette	5	0.34	0.25 ~ 0.44	0%	0 ~ 64	**<.001**
Total between						.19

*N*_*comp*_ Number of comparisons, *p*^a^: Values indicating the difference within subgroups; Total between^b^: *p* value indicating the difference between the sub groups

^a)^ The 95% *PI* cannot be calculated when the number of studies is lower than 3

^b)^ The 95% *CI* of *I*^2^ cannot be calculated when the number of studies is lower than 3

**Table 4:** Stigma for CMDs between racial minorities and majorities: Effect sizes of primary outcomes in all studies

Study	*Ethnic Group*	*Type*	*Outcomes*	*g*	95% *CI*	Forest plot of Hedges’ g and 95% CI
Adewuya, 2008 [31]	Other	SR	SDS	-0.15	-0.36~0.05	
Ahn 2015 [32]	Asian	SR	PDD	-0.12	-0.21~-0.03	
Anglin 2016 [18]	Black	VIG	Study	-0.13	-0.32~0.05	
Aznar-Lou 2016 [33]	Asian	SR	CAMI-23	0.67	-0.06~1.42	
Aznar-Lou 2016 [33]	Black	SR	CAMI-23	0.15	-0.11~0.41	
Aznar-Lou 2016 [33]	Other	SR	CAMI-23	0.17	-0.07~0.41	
Brown 2010 [19]	Black	SR	ISMI, PDD	-0.07	-0.25~0.11	
Caplan 201 1[22]	Hisp.	SR	Study	-0.04	-0.33~0.24	
Cheng 2015 [15]	Asian	VIG	AQ	0.29	0.10~0.48	
Conner 2010 [34]	Black	SR	ISMI, PDD	0.16	-0.02~0.35	
Conner 2009 [35]	Black	SR	ISMI, PDD	0.76	0.36~1.17	
Copelj 2011 [36]	Other	VIG	DSS	1.04	0.64~1.44	
Eisenberg 2009 [37]	Asian	SR	PDD	0.16	0.07~0.24	
Eisenberg 2009 [37]	Black	SR	PDD	0.51	0.39~0.64	
Eisenberg 2009 [37]	Hisp.	SR	PDD	0.15	0.04~0.27	
Eisenberg 2009 [37]	Other	SR	PDD	0.17	0.04~0.29	
Fogel 2005 [16]	Asian	SR	Study	0.31	0.26~0.35	
Georg Hsu 2008 [17]	Asian	VIG	Study	0.67	0.27~1.07	
Givens 2007 [21]	Native	SR	Study	-0.21	-0.27~-0.14	
Givens 2007 [21]	Hisp.	SR	Study	-0.01	-0.05~0.02	
Hickie 2007 [34]	Asian	SR	Study	0.33	-0.01~0.68	
Jimenez 2012 [23]	Black	SR	Study	0.16	0.05~0.26	
Makowski 2017 [11]	Other	VIG	Study	0.15	-0.00~0.31	
Menke 2009 [38]	Black	SR	LSCS	0.23	0.05~0.41	
Mokkarala 2016 [39]	Asian	SR	Study	0.21	-0.09~0.52	
Nadeem 2007 [8]	Black	SR	Study	0.16	-0.09~0.52	
Nadeem 2007 [8]	Hisp.	SR	Study	0.13	0.01~0.24	
O`Mahen 2011 [40]	Black	SR	LSCS	0.37	0.20~0.54	
Papadopoulos 2002 [41]	Other	SR	CAMI-23	0.51	0.09~0.24	
Picco 2016 [42]	Asian	SR	ISMI	-0.14	0.20~0.81	
Rao 2007 [43]	Asian	VIG	AQ	0.47	0.19~0.75	
Rao 2007 [43]	Hispanic	VIG	AQ	0.42	0.17~0.67	
Rüsh 2012 [56]	Asian	SR	CAMI-23	0.21	0.11~0.45	
Rüsh 2012 [44]	Black	SR	CAMI-23	0.28	0.22~0.89	
Schafer 201 1[45]	Black	SR	CAMI-23	0.56	0.16~0.54	
Shamblaw 2015 [46]	Asian	SR	DAQ, SDS	0.35	-0.23~-0.06	
Subramaniam 2017 [23]	Asian	VIG	DSS	-0.14	0.08~0.23	
Wang 2013 [47]	Black	VIG	SDS	0.36	0.20~0.53	
Wang 2013 [47]	Hisp.	VIG	SDS	0.15	-0.12~0.42	

*HISP* Hispanic, *SR* Self-Report, *VIG* Vignette, *Study* Study-constructed questionnaire

**Fig. 3:**
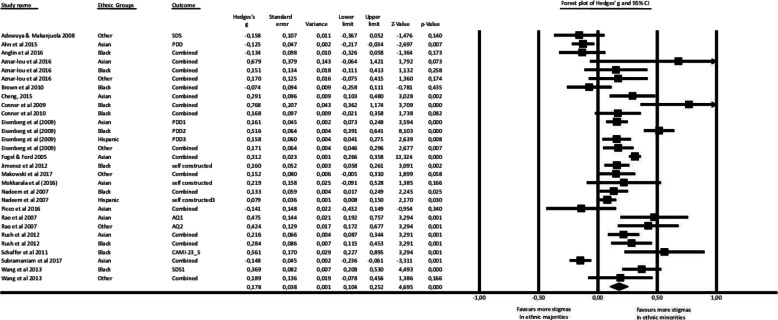
Stigma for CMDs between ethnic minorities and majorities: Forest plot when low quality studies excluded

**Fig. 4:**
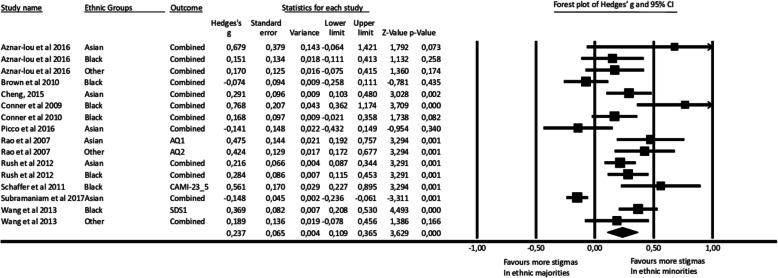
Stigma for CMDs between ethnic minorities and majorities: Forest plot when only studies with standardized outcome measures are included

**Fig. 5:**
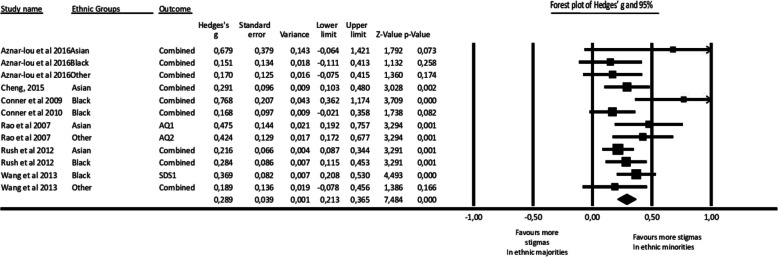
Stigma for CMDs between ethnic minorities and majorities: Forest plot when outliers are excluded

When studies with high risk of bias (i.e. defined as those with high risk of bias scores on three and/or more categories of the risk of bias assessment tool) were excluded, the effect was sustained g = 0.20 (95% CI: 0.10 ~ 0.25) and the heterogeneity was still very high (*I*^2^ = 88, 95% CI: 84 ~ 90) (see figure 3 in Appendix 4 for the forest plot). When only studies with standardized outcome measures were included, the effect size was slightly greater g = 0.23 (95% CI: 0.10 ~ 0.36) and the heterogeneity remained high (*I*^2^ = 84, 95% CI: 75 ~ 89) (see figure 4 in Appendix 5 for the forest plot). Next, excluding outliers resulted in a comparable effect size g = 0.29 (95% CI: 0.21 ~ 0.36) and less heterogeneity (*I*^2^ = 29, 95% CI: 0 ~ 63) (see Table 5 and figure 5 in Appendix 6 for the forest plot). To identify outliers, we looked at whether the 95% CI of the study overlaps with the 95% CI of the pooled effects size and we also looked whether a study considerably differs from the other included studies in the metanalysis [57]. Even though the CI of the study by Subramaniam and colleagues [22] did fall between the CI of the pooled effect sizes, the study did conceptualise stigma differently compared to the other studies. Additionally, excluding that study reduced the heterogeneity significantly and therefore we decided to exclude it (see Table 3).

**Table 5:** Stigma for CMDs between racial minorities and majorities: Forest plot when outliers are excluded

Study	*Ethnic Group*	*Type of Study*	*Outcomes*	*g*	95% *CI*	g (95% CI)
Aznar-Lou 2016 [33]	Asian	Self-report	CAMI-23	0.67	-0.06~1.42	
Aznar-Lou 2016 [33]	Black	Self-report	CAMI-23	0.15	-0.11~0.41	
Aznar-Lou 2016 [33]	Other	Self-report	CAMI-23	0.17	-0.07~0.41	
Cheng 2015 [15]	Asian	Vignette	AQ	0.29	0.10~0.48	
Conner 2010 [34]	Black	Self-report	ISMI, PDD	0.16	-0.02~0.35	
Conner 2009 [34]	Black	Self-report	ISMI, PDD	0.76	0.36~1.17	
Rao 2007 [43]	Asian	vignette	AQ	0.47	0.19~0.75	
Rao 2007 [43]	Hispanic	vignette	AQ	0.42	0.17~0.67	
Rüsh 2012 [44]	Asian	Self-report	CAMI-23	0.21	0.11~0.45	
Rüsh 2012 [44]	Black	Self-report	CAMI-23	0.28	0.22~0.89	
Wang 2013 [47]	Black	vignette	SDS	0.36	0.20~0.53	
Wang 2013 [47]	Hispanic	vignette	SDS	0.15	-0.12~0.42	

Egger’s test did not indicate a significant publication bias (*p* = 0.100). Duvall and Tweedie’s trim and fill procedure indicated 2 missing studies (see the funnel plot with imputed studies in Appendix 7). The adjusted effect size (after imputation of the missing studies) was g = 0.27 (95% CI: 0.20 ~ 0.33).

We did not investigate whether there was a variability in stigma perspectives among racial minorities in the subgroup and in multi-variate analyses since the remaining number of studies in some categories (perceived stigma *N* = 2, stigmatized *N* = 1) were not sufficient. No significant differences were found in subgroup analyses (see Table 2). Multi-variate analyses indicated no significant associations between the effect size and the racial groups (*p* = 0.42), quality (*p* = 0.12) and the type of the outcome of the studies (self-report, vignette) (*p* = 0.48) (see Table 6). However, there was a significant association between studies with high risk of bias and the effect size of stigma (*p* = 0.04).

**Table 6:** Multi-variate meta-regression analyses of predictors of stigma, by quality of studies, ethnicity and type of stigma outcomes in 10 studies of stigma in ethnic minorities and majorities

*Characteristics*	*N*	*SE*	*β*	*95% CI*	*Z*	*P*
Stigma Outcome						
Self-report	7	0.09	0.14	−0.17 ~ 0.37	0.70	.48
Vignette (ref)	5					
Quality of the Studies						.11
Weak	5	0.13	0.27	0.01 ~ 0.53	2.07	**.04**
Strong	2	0.11	−0.03	−0.25 ~ 0.18	−0.34	.73
Moderate (ref)	5					
Ethnicity						.42
Asian	4	0.12	0.28	−0.08 ~ 0.41	1.31	.19
Black	5	0.10	0.53	−0.11 ~ 0.29	0.83	.40
Other (ref)	3					

*Point Est* Point Estimate, *p* values indicating the difference between the effect sizes in subgroups, *ref* reference group

## Discussion

This systematic review and meta-analysis was aimed at comparing mental illness stigma associated with CMDs based on racial background. In line with our expectations, the results suggest that racial minorities have more mental illness stigma for CMDs when compared with racial majorities. Sensitivity analyses showed the robustness of these results. The multi-variate meta-regression analyses indicated that studies of poor quality had higher effect sizes than the studies with high quality.

Higher mental illness stigma in racial minorities is in line with the growing literature highlighting the variations in mental illness stigma based on ethnicity and/or race [11, 12, 56, 18–23]. This finding could be explained in relation to the social identity theory [58]. In collectivistic cultures, group harmony and cohesion are of central importance [24]. In this context, CMDs might be seen to fall outside of societal expectations [13, 58, 59] and this would precipitate shame and subsequent mental-illness stigma [58, 59]. Thus, what is defined as “them” not “us”, would reinforce the public opinion that individuals with CMDs are dangerous and must be segregated [60]. Consistent with this notion, there is research evidence indicating that cultural beliefs of CMDs are related with the extent of the impact of mental illness stigma (i.e. fear of someone with CMDs and/or desire for social distance from an individual with CMDs) at individual and societal levels [24, 60–63].

In extent, the consequences of mental illness stigma are more harmful especially when the preceding cultural and/or personal attributions coincide with social adversities such as migration, poverty, gender, diagnosis of a CMD, ethnic and/or sexual minority statuses [7–9, 63, 64]. This phenomenon is known as the intersectional impact of stigma and it accounts for the underutilisation of mental health services among those who are affected [10, 63–67]. For instance, a large scale study from America (*N* = 15,383) showed that, Black women with low socio-economic background reported more stigma related concerns and were less likely to utilise mental health services compared to locally born women from White backgrounds [8]. Furthermore, the negative impact of the intersection of different types of stigma on utilisation of health services is well-documented in the HIV literature investigating (HIV-related stigma, sexism, racism, and homo/transphobia) on individuals` well-being [65–67].

Studies comparing public beliefs and attitudes regarding CMDs point to the importance of migration status and/or ethnicity in shaping mental illness stigma [23]. For instance, in a large scale vignette study (*N* = 2013) by Makowski and colleagues in Germany, there was an indication for the differences in mental illness stigma between locally born minorities, migrants and non-minorities [23]. When the vignette concerned an individual from a migrant background with depression, participants from migrant background expressed greater stigmatizing attitudes (e.g. negative stereotypes, emotional reactions and desire for social distance) compared to the locally born minorities and majorities [23].

### Strengths and limitations

This study has various innovations. This is the first meta-analysis in the stigma field utilizing a unified conceptual framework to pool the studies. This is an important step to advance the current understanding of the mental illness stigma and how racial/ethnic differences impact people’s experiences of mental illness stigma.

Based on the current results an important message for the public health field is to tailor the existing anti-stigma interventions according to the specific racial and/or ethnic groups [25]. At present, recommended practices include psycho-educational campaigns aiming to improve the public knowledge on CMDs and individuals with CMDs [25, 68]. Such practices however, fail to demonstrate changes in attitudes among stigmatized and stigmatizer [69]. Often, educational campaigns do not provide evidence for the effectiveness of anti-stigma campaigns in increasing help-seeking for CMDs [69]. Our results suggest that the development and the implementation of such campaigns can be improved if the messages of these campaigns are adapted according to the socio-cultural and political contexts of the countries that the individuals live in [69, 70].

Even though our results suggest that all racial minorities, regardless, have higher mental illness stigma, various limitations need to be taken into account whilst interpreting our findings. We found no variability in mental illness stigma based on specific races. This could be explained with the small number of studies representing each racial minority group in sub-group analyses. Further, we were also unable to investigate if there were differences in mental illness stigma between racial minorities based on the migration status since this was rarely investigated in the included studies [8, 23]. It may be that there are other potential moderators such as the degree of acculturation which are overlooked in the current stigma literature and could not be taken into consideration in our study.

Further, the multi-variate analyses pointed to the possible moderating role of the number of studies with high risk of bias on the effect size of stigma. It could be that if there were more studies with low risk of bias, we might have found no racial differences in stigma for CMDs. Given these reasons, the results of this study must be treated with caution.

The studies from North America were overrepresented in the current study. This limits our understanding of how contextual differences between continents influence the impact of mental illness stigma. For instance, the experiences of discrimination related with stigma might be different in Europe compared to America [23].

Related issues were the racial classification and the definition of minority and/or majority statuses in this study. Since there were many ethnic and religious groups in the included studies, we decided that the mutually exclusive category approach [28], re-allocating individuals in existing categories defined by the previous research [29], would be the most suitable. We have also defined minority and majority statuses based on the country of the included studies. Since the definition of race-ethnicity includes history, religion, language and socio-political dynamics [28, 70], we oversimplified the term by using broad racial categories. Moreover, our definitions do not capture the changes in ethnicity and minority statuses over time depending on the changing socio-political circumstances [28]. An alternative to the preceding is the multiple characteristics approach [28] taking into account the various aspects of ethnicity such as language, country of birth, nationality and religiosity [28]. Even though the latter offers more effective approach to the measurement of ethnicity in detail, the former is more pragmatic and facilitates the comparability of the studies across countries [28]. In the light of these caveats, our results are restricted with the broad racial categories which are defined at a specific point in time. It is recommended that ethnicity and minority statuses are measured at different time points in prospective studies [28].

Another limitation restricting the representativeness of the sample in our study was the exclusion of clinical populations that had comorbidities. One reason could be that we have excluded HIV, and other comorbidities and yet most clinical studies include people who often have CMDs and other conditions. Some studies indicate that diagnosis of a CMD would reinforce the label indicating that the individual falls out of the societal expectations and therefore is unpredictable and/or dangerous [8, 46, 71]. Alternatively, diagnosis of a CMD might create opportunities for more psycho-education about CMDs, and more contact with the others with CMDs and this might reduce the mental illness stigma in return [71]. Since clinical populations are not represented in our study, we were not able to assess if there was a variation in mental illness stigma among racial minorities depending on the presence of a formal diagnosis. Thus, the following questions are important and yet they remain to be answered by the future studies: Among those who have received a diagnosis of a CMD, are there any differences in the experience of mental illness stigma between ethnic minorities and majorities? Among ethnic minorities, are there differences in mental illness stigma between those who have been diagnosed with CMDs and those who have not?

Further, some types of mental-illness stigma are under investigated. There is an indication that stigma is associated with greater burden when it is accepted and internalized by those who have a mental illness [11]. In our meta-analysis, many studies investigated stereotypes or prejudices attributed to those who have a mental illness. Conversely, how stigma was anticipated or experienced by ethnic minorities when compared with majorities were rarely investigated [20, 21]. Given this limitation, we could not investigate whether there was a variation in different perspectives (e.g. anticipated, internalised) and/or components (e.g. cognitive, affective, behavioural) of mental illness stigma between racial minorities.

It is worth to emphasize that there were considerable number of the questionnaires which were developed for the purpose of the studies by the authors themselves. In our sensitivity analyses, exclusion of these questionnaires and limiting the analyses with the validated questionnaires such as CAMI, reduced the heterogeneity. The revision of the existing self-report questionnaires by the authors was another limitation. Often, these questionnaires were revised according to the research question and/or the sample and their psychometric properties were not tested. Additionally, the self-report questionnaires often measured multiple stigma mechanisms within the same scale [11]. This might conflate the results as stigma mechanisms might differently relate to the outcomes.

Poor quality of the vignette studies also worth mentioning. The vignettes were developed based on the DSM criteria and the authors` clinical information of the CMDs. Often, there was no information about the psychometric properties of the vignettes with one exception. Subramaniam and colleagues followed a systematic approach by revising the vignettes with clinical experts, piloting them with participants and revising the relevance and acceptability afterwards [23]. Since the vignettes are often developed by the researchers themselves, it could be that they are more suggestive when compared with self-report measures and this would confound the results (see Appendix 2).

Our results are also limited with the MISF as other frameworks exist such as the Framework Integrating Normative Influences on Stigma (FINIS) [72]. We have chosen the MISF as to our knowledge, it identifies specific outcome measures capturing specific stigma mechanism [11].

## Conclusions

The limitations of our study underscore the importance of investigating the intersection of race/ethnicity, degree of acculturation, presence of a mental illness diagnosis and the impact of mental health stigma. There is a need for more high quality research for the advancement of the stigma field. The quality of the future studies could be improved by defining meaningful controls. For instance, researchers could relate sample characteristics to the general population of the country of the study. Additionally, the racial and or ethnic groups of the country of the studies should be represented. In line with the multiple characteristics approach to define ethnicity, future studies could define the ethnic composition of their sample consistently based on the characteristics such as history and religion which are outlined earlier. Furthermore, more prospective studies are needed to capture the changes in ethnic classification over time. Prospective studies are also crucial to examine whether the degree of acculturation, diagnosis of a CMD and/or minority statuses are effect modifiers in the relationship between ethnicity and mental illness stigma.

To conclude, mental illness stigma is one of the important myriad of factors that might underpin individuals` state of physical, psychological, and social wellbeing. The results of the current meta-analysis indicate differences in mental illness stigma based on racial background and this result highlights the important role of racial and/ethnic background in shaping the mental illness stigma. An important clinical implication of these findings would be to tailor anti-stigma strategies according to the specific racial and/or ethnic backgrounds with the intention to improve mental health outreach [25].

## Figures and Tables

**Fig. 6:**
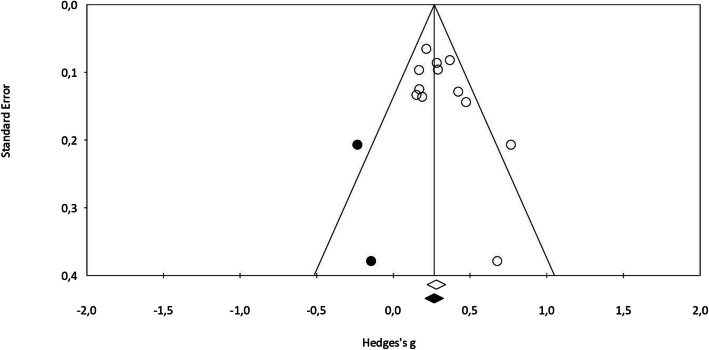
Stigma for CMDs between ethnic minorities and majorities: Funnel Plot

## Data Availability

The dataset of the current study is available from the corresponding author on reasonable request.

## Appendix 1

**Table:**

**Databases searched**	**Search Results 20 December 2018**
**PubMed (789 items)**	("Social Stigma"[Mesh] OR stigma*[tiab] OR mental health belief*[tiab] OR patient belief*[tiab] OR patients belief*[tiab]) AND ("Transients and Migrants"[Mesh] OR "Emigrants and Immigrants"[Mesh] OR "Ethnic Groups"[Mesh] OR "Minority Groups"[Mesh] OR "Minority Health"[Mesh] OR "Refugees"[Mesh] OR "Refugee Camps"[Mesh] OR immigrant*[tiab] OR migrant*[tiab] OR ethnic*[tiab] OR minorit*[tiab] OR non-western[tiab] OR nonwestern[tiab] OR refug*[tiab] OR asylum[tiab]) AND ("Mental Disorders"[Mesh:noexp] OR "Stress, Psychological"[Mesh] OR "Mood Disorders"[Mesh:NoExp] OR "Anxiety Disorders"[Mesh] OR "Diagnostic and Statistical Manual of Mental Disorders"[Mesh] OR "Anxiety"[Mesh:NoExp] OR "Depression"[Mesh] OR "Depressive Disorder, Major"[Mesh] OR "Dysthymic Disorder"[Mesh] OR psychological distress[tiab] OR psychological stress[tiab] OR anxiety[tiab] OR anxieties[tiab] OR anxious[tiab] OR depress*[tiab] OR dysthymi*[tiab] OR phobi*[tiab] OR panic[tiab] OR common mental disorder*[tiab] OR "Stress Disorders, Traumatic"[Mesh:NoExp] OR "Stress Disorders, Post-Traumatic"[Mesh] OR "Stress Disorders, Traumatic, Acute"[Mesh] OR Acute Stress Disorder*[tiab] OR Post-Traumatic Stress Disorder*[tiab] OR PTSD[tiab] OR mood disorder*[tiab])
**Embase (1015 items)**	('social stigma'/exp OR stigma*:ab,ti,kw OR 'mental health belief*':ab,ti,kw OR 'patient belief*':ab,ti,kw OR 'patients belief*':ab,ti,kw) AND ('migration'/exp OR 'migrant'/exp OR 'ethnic group'/exp OR 'minority group'/exp OR 'minority health'/exp OR 'refugee camp'/exp OR immigrant*:ab,ti,kw OR migrant*:ab,ti,kw OR ethnic*:ab,ti,kw OR minorit*:ab,ti,kw OR 'non-western':ab,ti,kw OR nonwestern:ab,ti,kw OR refug*:ab,ti,kw OR asylum:ab,ti,kw) AND ('mental disease'/de OR 'mental stress'/exp OR 'mood disorder'/de OR 'anxiety disorder'/exp OR 'diagnostic and statistical manual of mental disorders'/exp OR 'anxiety'/de OR 'depression'/de OR 'dysthymia'/exp OR 'major depression'/exp OR 'major affective disorder'/exp OR 'psychological distress':ab,ti,kw OR 'psychological stress':ab,ti,kw OR anxiety:ab,ti,kw OR anxieties:ab,ti,kw OR anxious:ab,ti,kw OR depress*:ab,ti,kw OR dysthymi*:ab,ti,kw OR phobi*:ab,ti,kw OR panic:ab,ti,kw OR 'common mental disorder*':ab,ti,kw OR 'acute stress disorder*':ab,ti,kw OR 'post-traumatic stress disorder*':ab,ti,kw OR ptsd:ab,ti,kw OR 'mood disorder*':ab,ti,kw)
**PsycINFO (846 items)**	( DE "Stigma" OR TI (stigma* OR "mental health belief*" OR "patient belief*" OR "patients belief*") OR AB (stigma* OR "mental health belief*" OR "patient belief*" OR "patients belief*") ) AND ( DE "Human Migration" OR DE "Refugees" OR DE "Asylum Seeking" OR DE "Immigration" OR DE "Racial and Ethnic Groups" OR DE "Minority Groups" OR TI (immigrant* OR migrant* OR ethnic* OR minorit* OR "non-western" OR nonwestern OR refug* OR asylum) OR AB (immigrant* OR migrant* OR ethnic* OR minorit* OR "non-western" OR nonwestern OR refug* OR asylum) ) AND ( DE "Mental Disorders" OR DE "Psychological Stress" OR DE "Diagnostic and Statistical Manual" OR DE "Anxiety Disorders" OR DE "Acute Stress Disorder" OR DE "Panic Disorder" OR DE "Phobias" OR DE "Post-Traumatic Stress" OR DE "Posttraumatic Stress Disorder" OR DE "Anxiety" OR DE "Affective Disorders" OR DE "Major Depression" OR DE "Dysthymic Disorder" OR DE "Depression (Emotion)" OR TI ("psychological distress" OR "psychological stress" OR anxiety OR anxieties OR anxious:ab,ti,kw OR depress* OR dysthymi* OR phobi* OR panic OR "common mental disorder*" OR "Acute Stress Disorder*" OR "Post-Traumatic Stress Disorder*" OR PTSD OR "mood disorder*") OR AB ("psychological distress" OR "psychological stress" OR anxiety OR anxieties OR anxious:ab,ti,kw OR depress* OR dysthymi* OR phobi* OR panic OR "common mental disorder*" OR "Acute Stress Disorder*" OR "Post-Traumatic Stress Disorder*" OR PTSD OR "mood disorder*") )

## Appendix 2

### Vignette and Manipulation

J.S. was a typical man who enjoyed going out to dinner after work with his coworkers, and playing sports on the weekend with his friends. Recently, he started feeling very down and unhappy. After he found it very hard to get out of bed, get dressed, go to work, or do anything for several weeks, he decided to go see a psychiatrist. The psychiatrist asked him about his symptoms, which included not getting any pleasure out of things he normally would and having trouble sleeping and eating. At times, he would also feel completely worthless and even had thoughts about killing himself.

Genetic condition:

Because of J.S.’s symptoms, the psychiatrist diagnosed him with depression and recommended that he enroll in treatment. Many experts and doctors also tested to see what had caused J.S.'s problem. After many assessments, the doctors found no imbalance of brain chemicals, and no significant childhood or recent events that could have contributed to the onset of depression. But experts in genetics did find a genetic predisposition and family history of depression, and the majority of doctors concluded that J.S.'s problem was largely due to J.S.'s genes (i.e., genetic factors).

Neurobiological condition:

Because of J.S.’s symptoms, the psychiatrist diagnosed him with depression and recommended that he enroll in treatment. Many experts and doctors also tested to see what had caused J.S.'s problem. After many assessments, the doctors found no genetic predisposition or family history of mental illness, and no significant childhood or recent events that could have contributed to the onset of depression. But experts in neurobiology did find an imbalance of brain chemicals and the majority of doctors concluded that J.S.'s problem was largely due to the imbalance of brain chemicals (i.e., neurobiological factors).

Social condition:

Because of J.S.’s symptoms, the psychiatrist diagnosed him with depression and recommended that he enroll in treatment. Many experts and doctors also tested to see what had caused J.S.'s problem.  After many assessments, the doctors found no genetic predisposition or family history of mental illness, and no imbalance of brain chemicals. But experts did find that J.S. had gone through many significant events, such as losing his best friend at a young age and a recent divorce that could have contributed to the onset of depression. The majority of doctors concluded that J.S.'s problem was largely due to his childhood and recent events (i.e., social factors).

Control condition:

Because of J.S.’s symptoms, the psychiatrist diagnosed him with depression and recommended that he enroll in treatment. Many experts and doctors also tested to see what had caused J.S.'s problem. After many assessments, the majority of doctors were not able to come to a decision about what had caused J.S.'s problem.

Correspondence should be addressed to Zhen H. Cheng, Department of Psychology, University of Oregon, Eugene, OR 97403. E-mail: zcheng@uoregon.edu

## Abbreviations

CMDs: Common Mental Disorders
MISF: Mental Illness Framework
EPHPP: Effective Public Health Practice Project Quality Assessment Tool
PDD: Perceived Devaluation and Discrimination Scale
ISMI: Internalized Stigma of Mental Illness Scale
CAMI: Community Attitudes to Mental Illness scale
CMA: Comprehensive Meta-Analysis
FINIS: Framework Integrating Normative Influences on Stigma
AQ: Attribution Questionnaire
SDS: Social Distance Scale
DSS: Depression Stigma Scale
DAQ: Depression Attribution Questionnaire
LSCS: Link Stigma Consciousness Scale
RoB: Risk of Bias Assessment
