# Correction to: Stigma for common mental disorders in racial minorities and majorities a systematic review and meta-analysis

**DOI:** 10.1186/s12889-020-09199-y

**Published:** 2020-09-01

**Authors:** Ozlem Eylem, Leonore de Wit, Annemieke van Straten, Lena Steubl, Zaneta Melissourgaki, Gözde Topgüloğlu Danışman, Ralph de Vries, Ad J. F. M. Kerkhof, Kamaldeep Bhui, Pim Cuijpers

**Affiliations:** 1 Department of Clinical, Neuro and Developmental Psychology, Amsterdam Public Health Research Institute, Vrije Universiteit Amsterdam, Van der Boechorststraat 1, 1081 BT Amsterdam, The Netherlands; 2 Centre for Psychiatry, Queen Mary University of London, London, UK; 3 Department of Clinical Psychology and Psychotherapy, University of Ulm, Ulm, Germany; 4 Department of Psychology, University of Warwick, Coventry, UK; 5 Faculty of Social Sciences, Centre for Family and Couple Therapy, Özyeğin University, İstanbul, Turkey; 6 Medical Library, Vrije Universiteit, Amsterdam, the Netherlands

**Correction to: BMC Public Health 20, 879 (2020)**

**https://doi.org/10.1186/s12889-020-08964-3**

It was highlighted that in the original article [1] Table 4 contained an error in its formatting. This Correction article shows the correct Table 4. The original article has been updated.

**Table 4:** Stigma for CMDs between racial minorities and majorities: Effect sizes of primary outcomes in all studies

Study	*Ethnic Group*	*Type*	*Outcomes*	*g*	95% *CI*	Forest plot of Hedges’ g and 95% CI
Adewuya, 2008 [31]	Other	SR	SDS	-0.15	-0.36~0.05	
Ahn 2015 [32]	Asian	SR	PDD	-0.12	-0.21~-0.03	
Anglin 2016 [18]	Black	VIG	Study	-0.13	-0.32~0.05	
Aznar-Lou 2016 [33]	Asian	SR	CAMI-23	0.67	-0.06~1.42	
Aznar-Lou 2016 [33]	Black	SR	CAMI-23	0.15	-0.11~0.41	
Aznar-Lou 2016 [33]	Other	SR	CAMI-23	0.17	-0.07~0.41	
Brown 2010 [19]	Black	SR	ISMI, PDD	-0.07	-0.25~0.11	
Caplan 2011[22]	Hisp.	SR	Study	-0.04	-0.33~0.24	
Cheng 2015 [15]	Asian	VIG	AQ	0.29	0.10~0.48	
Conner 2010 [34]	Black	SR	ISMI, PDD	0.16	-0.02~0.35	
Conner 2009 [18]	Black	SR	ISMI, PDD	0.76	0.36~1.17	
Copelj 2011 [35]	Other	VIG	DSS	1.04	0.64~1.44	
Eisenberg 2009 [36]	Asian	SR	PDD	0.16	0.07~0.24	
Eisenberg 2009 [36]	Black	SR	PDD	0.51	0.39~0.64	
Eisenberg 2009 [36]	Hisp.	SR	PDD	0.15	0.04~0.27	
Eisenberg 2009 [36]	Other	SR	PDD	0.17	0.04~0.29	
Fogel 2005 [16]	Asian	SR	Study	0.31	0.26~0.35	
Georg Hsu 2008 [17]	Asian	VIG	Study	0.67	0.27~1.07	
Givens 2007 [21]	Native	SR	Study	-0.21	-0.27~-0.14	
Givens 2007 [21]	Hisp.	SR	Study	-0.01	-0.05~0.02	
Hickie 2007 [37]	Asian	SR	Study	0.33	-0.01~0.68	
Jimenez 2012 [23]	Black	SR	Study	0.16	0.05~0.26	
Makowski 2017 [11]	Other	VIG	Study	0.15	-0.00~0.31	
Menke 2009 [38]	Black	SR	LSCS	0.23	0.05~0.41	
Mokkarala 2016 [39]	Asian	SR	Study	0.21	-0.09~0.52	
Nadeem 2007 [8]	Black	SR	Study	0.16	-0.09~0.52	
Nadeem 2007 [8]	Hisp.	SR	Study	0.13	0.01~0.24	
O`Mahen 2011 [40]	Black	SR	LSCS	0.37	0.20~0.54	
Papadopoulos 2002 [41]	Other	SR	CAMI-23	0.51	0.09~0.24	
Picco 2016 [42]	Asian	SR	ISMI	-0.14	0.20~0.81	
Rao 2007 [43]	Asian	VIG	AQ	0.47	0.19~0.75	
Rao 2007 [43]	Hispanic	VIG	AQ	0.42	0.17~0.67	
Rüsh 2012 [44]	Asian	SR	CAMI-23	0.21	0.11~0.45	
Rüsh 2012 [44]	Black	SR	CAMI-23	0.28	0.22~0.89	
Schafer 2011 [45]	Black	SR	CAMI-23	0.56	0.16~0.54	
Shamblaw 2015 [46]	Asian	SR	DAQ, SDS	0.35	-0.23~-0.06	
Subramaniam 2017 [23]	Asian	VIG	DSS	-0.14	0.08~0.23	
Wang 2013 [47]	Black	VIG	SDS	0.36	0.20~0.53	
Wang 2013 [47]	Hisp.	VIG	SDS	0.15	-0.12~0.42	

Note. *HISP* Hispanic, *SR* Self-Report, *VIG* Vignette, *Study* Study-constructed questionnaire

## References

[1] Eylem (2020). Stigma for common mental disorders in racial minorities and majorities a systematic review and meta-analysis. BMC Public Health.

